# Incidence, clinical features, and outcomes of posterior circulation ischemic stroke: insights from a large multiethnic stroke database

**DOI:** 10.3389/fneur.2024.1302298

**Published:** 2024-02-07

**Authors:** Yahia Z. Imam, Prem Chandra, Rajvir Singh, Ishrat Hakeem, Sally Al Sirhan, Mona Kotob, Naveed Akhtar, Saadat Kamran, Salman Al Jerdi, Ahmad Muhammad, Khawaja Hasan Haroon, Suhail Hussain, Jon D. Perkins, Osama Elalamy, Mohamed Alhatou, Liaquat Ali, Mohamed S. Abdelmoneim, Sujatha Joseph, Deborah Morgan, Ryan Ty Uy, Zain Bhutta, Aftab Azad, Ali Ayyad, Ahmed Elsotouhy, Ahmed Own, Dirk Deleu

**Affiliations:** ^1^Neuroscience Institute, Hamad Medical Corporation, Doha, Qatar; ^2^Weill Cornell Medicine-Qatar, Doha, Qatar; ^3^College of Medicine, Qatar University, Doha, Qatar; ^4^Statistics, Medical Research Center, Hamad Medical Corporation, Doha, Qatar; ^5^Cardiology Research Center, Hamad Medical Corporation, Doha, Qatar; ^6^Department of Emergency Medicine, Hamad Medical Corporation, Doha, Qatar

**Keywords:** observational, incidence, clinical features, posterior circulation stroke, multiethnic, outcomes

## Abstract

**Background:**

Posterior cerebral circulation ischemic stroke (PCS) comprises up to 25% of all strokes. It is characterized by variable presentation, leading to misdiagnosis and morbidity and mortality. We aim to describe PCS in large multiethnic cohorts.

**Methods:**

A retrospective review of a large national stroke database from its inception on the 1st of January 2014 till 31 December 2020. Incidence per 100,000 adult population/year, demographics, clinical features, stroke location, and outcomes were retrieved. We divided the cohort into patients from MENA (Middle East and North Africa) and others.

**Results:**

In total, 1,571 patients were identified. The incidence of PCS was observed to be rising and ranged from 6.3 to 13.2/100,000 adult population over the study period. Men were 82.4% of the total. The mean age was 54.9 ± 12.7 years (median 54 years, IQR 46, 63). MENA patients comprised 616 (39.2%) while others were 954 (60.7%); of these, the majority (80.5%) were from South Asia. Vascular risk factors were prevalent with 1,230 (78.3%) having hypertension, 970 (61.7%) with diabetes, and 872 (55.5%) having dyslipidemia. Weakness (944, 58.8%), dizziness (801, 50.5%), and slurred speech (584, 36.2%) were the most commonly presenting symptoms. The mean National Institute of Health Stroke Score (NIHSS) score was 3.8 ± 4.6 (median 3, IQR 1, 5). The overall most frequent stroke location was the distal location (568, 36.2%). The non-MENA cohort was younger, less vascularly burdened, and had more frequent proximal stroke location (*p* < 0.05). Dependency or death at discharge was seen in 39.5% and was associated with increasing age, and proximal and multilocation involvement; while at 90 days it was 27.4% and was associated with age, male sex, and having a MENA nationality (*p* < 0.05).

**Conclusion:**

In a multiethnic cohort of posterior circulation stroke patients from the MENA region and South Asia, we noted a rising incidence over time, high prevalence of vascular risk factors, and poor outcomes in older men from the MENA region. We also uncovered considerable disparities between the MENA and non-MENA groups in stroke location and outcome. These disparities are crucial factors to consider when tailoring individualized patient care plans. Further research is needed to thoroughly investigate the underlying reasons for these variations.

## Introduction

Posterior cerebral circulation ischemic stroke constitutes approximately 20%–25% of all ischemic stroke (1). It poses unique challenges to treating clinicians in that it has variable, nonspecific presentation owing to the vast area of supply, the complexity of the structures supplied, and the significant anatomical variations observed (2). Furthermore, even a score of zero on the National Institute of Health Stroke Scale (NIHSS) is not synonymous with “no stroke” given that it is heavily biased to detect anterior circulation stroke (3, 4). In addition, plain CT head scans are not sensitive enough which can lead to a false sense of security leading it to be frequently missed (5). This predisposes PCS patients to significant morbidity and mortality with basilar thrombosis in particular a feared presentation.

Most of the descriptions of PCS have come from registry data and selections of posterior circulation patients from large clinical trials or multicenter cohorts (6–8), however, PCS is largely overshadowed by the more common anterior circulation stroke (ACS). Zhang et al. (8) reported on 690 patients from 22 Chinese centers with noncardiogenic PCS due to intracranial atherosclerotic disease (ICAD) and provided insights indicating that PCS due to non-vertebral ICAD was more common than vertebral ICAD in the Chinese population. However, the latter had more serious clinical-radiologic patterns and worse outcomes.

A large Iranian cohort study (9) on stroke compared ACS to PCS. It is noteworthy that the cohort had only 76 patients with PCS. It concluded that there was no difference in mortality and functional outcome between the two groups.

Another important registry is the New England Medical Center Posterior Circulation Registry (NEMC-PCR) (6). It is a multiethnic, predominantly white database that has provided a widely used neuroimaging-based PCS classification. However, non-white patients have been underrepresented.

Different populations have different demographics and psychosocial and economic connotations that impact how disease is diagnosed and treated (10). Furthermore, there is mounting evidence of the presence of ethnic disparity in stroke incidence (11), stroke care, and outcome (12). The Middle East and North Africa plus region (MENA+) is known to have a high stroke burden and, more worryingly, at a younger age (13). Emerging reports suggest existing disparity in the region itself in terms of stroke facilities, access to care, and availability of acute therapies (14), therefore, it is important to shed light on such data to help inform future research.

In this current descriptive study, we aim to mainly describe PCS demographics, risk factors, clinical features, and outcomes in a large multiethnic database with a predominance of patients from the Middle East, North Africa, and South and Southeast Asia.

## Methods

This is a retrospective descriptive review of a large national stroke database from its inception on 1st January 2014 until 31 December 2020. Posterior cerebral circulation ischemic stroke (PCS) refers to a clinical syndrome resulting from ischemia caused by stenosis, *in-situ* thrombosis, or embolic occlusion affecting arteries in the posterior circulation. These arteries include the vertebral arteries in the neck, the intracranial vertebral, basilar, and posterior cerebral arteries, as well as their branches (15).

Patient inclusion criteria were adapted from Akhtar et al. (16) as:

1. Acute neurologic deficit attributable to posterior circulation and basilar artery occlusion, proven by computed tomographic angiogram (CTA), MRA, or conventional angiogram.
2. Acute neurologic deficit causing alternating hemi/tetraplegia with brain stem signs, visual loss, locked in state, coma, or death.
3. Coma or loss of consciousness and acute posterior circulation infarction on CT or MRI.

The Qatar Stroke database has a unique setup as Qatar has only one accredited comprehensive stroke center (Hamad General Hospital) and >90% of cases are either admitted, referred, or followed up here and are referenced in this prospective registry (17). Incidence per 100,000 adult population/year is calculated using the number of PCS/years divided by the total population from census data as previously described (18) or derived from the mid-year population estimates provided by the Planning and Statistics Authority (19).

The patients were grouped according to their nationality as follows. The Middle East and North Africa (MENA) region was defined as per the United Nations International Children’s Emergency Fund (UNICEF) definition (20). Whereas geographic regions within Asia were subdivided into East Asia, South Asia, and Southeast Asia according to United Nations Geoscheme (21). However, Iran was included in MENA as per the UNICEF definition as it shares tight geosocial ties with the rest of the MENA region. All other nationalities were included in the others.

### Risk factors

Diabetes mellitus (DM) was defined as per American Diabetes Association criteria (22), and dyslipidemia (23), hypertension (24), coronary artery disease (25), and atrial fibrillation (26) were defined as per these respective guidelines.

### Neuroimaging

This was described previously (16) and includes brain-imaging studies with CT and/or MRI (at least one), along with vascular imaging CTA or MRA or conventional angiogram.

### Clinical presentation, stroke location, and outcome data

**Figure 1:**
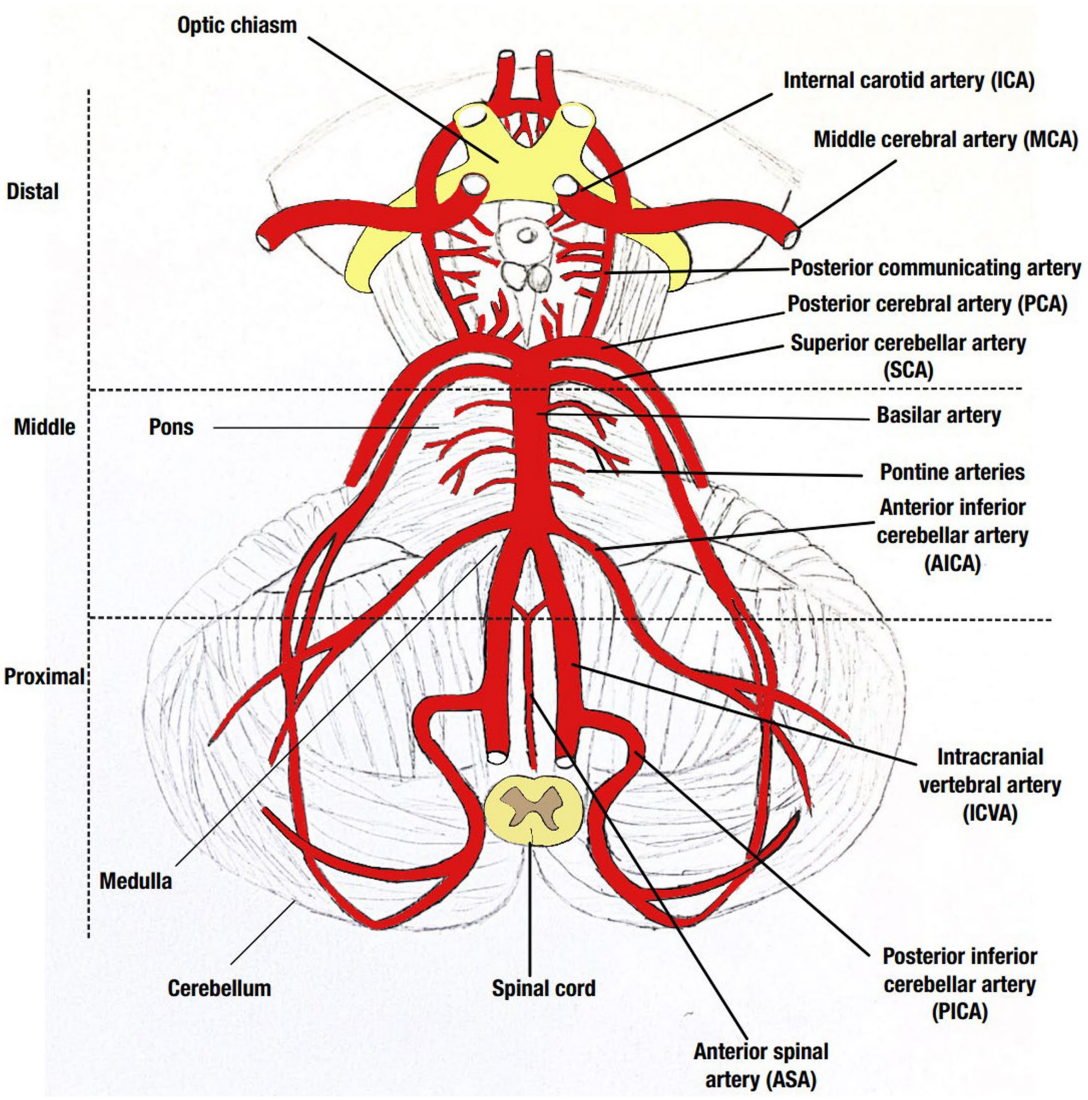
Illustration of the posterior circulation including the three distinct locations described in the New England Medical Center Posterior Circulation registry classification. Drawing by Sally Al-Sirhan adopted from Netter (28).

Presenting symptoms and signs were described. Stroke severity was assessed by the National Institute of Health Stroke Scale (NIHSS) (27) and the stroke location on neuroimaging was described using the New England Medical Center Posterior Circulation registry classification (6) as illustrated in Figure 1 (adapted from Frank Netter’s Atlas of Human Anatomy) (28). Stroke etiology was classified according to the TOAST classification (29). Outcomes were assessed at discharge and 90 days using the Modified Rankin Score (mRS) (30). It was dichotomized to show independence (mRS 0–2) or dependence/death (mRS 3–6).

### Statistical analysis

Descriptive statistics were used to summarize demographics, clinical features, neuroimaging findings, and outcomes of posterior circulation ischemic stroke cases and related risk factors. Quantitative results were reported with the mean and the standard deviation (SD), while the median and the interquartile range (IQR) were used for skewed/non-normal data distribution. Quantitative data (such as age, NIHSS, and mRS score) between MENA vs. Others were analyzed using an independent t test or the Mann–Whitney U test as appropriate. Incidence rates of PCS per 100,000 adult population by year were estimated and presented with respective 95% confidence intervals (CI). Confidence intervals for incidence rates were calculated using the OpenEpi software (31). Categorical data were summarized using frequencies and percentages. Associations between two or more categorical data were assessed using Chi-square (χ2) and/or Fisher Exact test as appropriate. Box plots were constructed to depict the distribution of age across outcome categories. All *p*-values presented were two-tailed, and values <0.05 were considered statistically significant. Statistical data analyses were performed using statistical software packages SPSS version 28.0 (Armonk, NY: IBM Corp) and Epi-info (Centers for Disease Control and Prevention, Atlanta, GA).

### Ethical consideration

This study was approved by the Institutional Review Board at the Medical Research Center, research protocol 03-SI-17-075.

## Results

A total of 1,571 PCS patients were identified. Of these 1,295 (82.4%) were men. The mean age of the PCS cohort was 54.9 ± 12.7 years (median 54 years, IQR 46, 63). Patients from South Asia constituted 768 (48.9%), followed by the MENA region with 616 (39.2%), then Southeast and East Asia with 145 (9.2%), and 42 from other regions (2.7%). The incidence of PCS ranged from 6.3 to 13.2/100,000 adult population as seen in Table 1.

**Table 1:** Incidence of posterior circulation strokes by year.

Year	Number of posterior circulation stroke cases	Qatar adult population^*^	Incidence rate (IR) per 100,000 population	95% confidence interval for IR
2014	126	1,852,027	6.80	5.72, 8.10
2015^*^	129	2,054,932	6.28	5.28, 7.46
2016	223	2,213,614	10.07	8.84, 11.49
2017	252	2,284,834	11.03	9.75, 12.48
2018	304	2,324,737	13.08	11.69, 14.63
2019	309	2,348,934	13.15	11.77, 14.71
2020^*^	228	2,325,816	9.80	8.61, 11.16

^*^Census data.

Vascular risk factors were prevalent, with 1,230 (78.3%) having hypertension, 970 (61.7%) with DM, 872 (55.5%) with dyslipidemia, 179 (11.4%) with coronary artery disease (CAD), 69 (4.4%) with atrial fibrillation (AF), and 184 (11.7%) had a prior history of Stroke/TIA.

The most prevalent presenting complaint was weakness (any of the face, arm, or leg or clumsiness) with 944 (58.8%), followed by 801 (50.5%) with dizziness, 584 (36.2%) with slurred speech, 207 (13.1%) with vertigo, 598 (28.8%) with nausea/vomiting, 450 (28.5%) with numbness, 428 (26.5%) with headache, and 365 (23.2%) with eye complaints including double vision, blurring of vision or field cut, as well as 196 (12.5%) with decreased sensorium and 164 (10.4%) with imbalance.

The NIHSS mean score was 3.8 ± 4.6 (median 3, IQR 1, 5). The most frequent location of stroke was the distal location with 568 (36.2%), followed by the proximal location with 464 (29.5%) and the least frequent was the middle location with 352 (22.4%). Multiple locations were observed in 187 (11.9%).

Demographics, stroke risk factors, stroke locations, and outcomes are illustrated in Table 2 and grouped into MENA vs. Others (non-MENA).

**Table 2:** Demographics, risk factors, stroke location, and outcomes in Middle East and North Africa (MENA) cohort *vs* non-MENA.

		MENA *N* = 616	Non-MENA *N* = 954	*p*-value
Age (years) (mean ± SD)		61.82 ± 13.16	50.46 ± 10.11	<0.001
Sex	Male	429 (69.6%)	866 (90.7%)	<0.001
	Female	187 (30.4%)	89 (9.3%)	
BMI (mean ± SD)		29.58 ± 5.91	26.85 ± 4.45	<0.001
DM		453 (80.3%)	517 (66.5%)	<0.001
HTN		504 (81.8%)	726 (76.1%)	0.007
Dyslipidemia		346 (56.4%)	526 (55.2%)	0.65
Smoking		146 (25.8%)	260 (29.8%)	0.105
CAD		101 (16.8%)	78 (8.3%)	<0.001
AF		40 (6.7%)	29 (3.1%)	<0.001
Prior stroke		116 (18.8%)	62 (6.5%)	<0.001
Prior TIA		4 (0.6%)	2 (0.2%)	0.17
Trauma		9 (1.5%)	8 (0.9%)	0.239
NIHSS (mean ± SD)		3.84 ± 4.73	3.83 ± 4.51	0.26
Thrombolysis		39 (6.3%)	71 (7.4%)	0.40
**Location**				
Proximal		137 (22.2%)	327 (34.2%)	<0.001
Middle		167 (27.1%)	185 (19.4%)	0.003
Distal		242 (39.3%)	326 (34.1%)	0.04
Proximal and distal		25 (4.1%)	39 (4.1%)	0.98
Proximal and Middle		13 (2.1%)	24 (2.5%)	0.61
Middle and distal		22 (3.6%)	23 (2.4%)	0.18
All 3		10 (1.6%)	31 (3.2%)	0.05
TOAST classification		*n* = 529 (89.5%)	*n* = 842 (88.3%)	
LAA		101 (19.1%)	170 (20.2%)	0.93
SVD		272 (51.4%)	423 (50.2%)	
CE		94 (17.8%)	141 (16.7%)	
OD		28 (5.3%)	51 (6.1%)	
UD		34 (6.4%)	57 (6.8%)	
Good outcome at discharge (mRS0-2)		366/614 (59.6%)	584/952 (61.3%)	0.492
Good outcome at 90 days (mRS0-2)		373/541 (68.9%)	554/735 (75.4%)	0.011
Death		75 (12.2%)	45 (4.7%)	<0.001

This is a retrospective data review study and for some parameters, the data values were incomplete due to the unavailability of the information in the patients’ record files and hence respective percentage (%) values were computed using non-missing data values. n, Number; SD, Standard Deviation; BMI, Body Mass Index; DM, Diabetes Miletus; HTN, Hypertension; CAD, Coronary Artery Disease; AF, Atrial Fibrillation; TIA, Transit Ischemic Attack; mRS, Modified Rankin Score; LAA, Large Artery Atherosclerosis; SVD, Small Vessel Disease; CE, Cardioembolic; OD, Other Determined; UD, Undetermined or Cryptogenic.

The non-MENA cohort was significantly younger and had significantly lower body weight, diabetes, hypertension, coronary artery disease, and atrial fibrillation (*p* < 0.05) than the MENA cohort.

The TOAST classification was available for 87.3% of patients. Small vessel disease (SVD, 50.7%) was the most frequent stroke etiology, followed by large artery atherosclerosis (LAA, 19.8%), and cardioembolic (CE, 17.1%). There was no significant difference observed (*p* > 0.05) in stroke etiology between the MENA and non-MENA contingents (Table 2).

The most prevalent locations among the proximal location were isolated PICA cerebellum involvement in 376 (62%) patients, whereas the pontine branches were the most prevalent in the middle location in 394 (82.9%) patients and PCA involvement in the distal location in 563 (78.4%) patients.

Over the study period (7 years) there were 49 recurrent events documented at the rate of 3.1%.

Outcome data was available for 100% of patients at discharge and 87.8% of the MENA cohort and 77% of the non-MENA group at 90 days. There were 120 fatalities at 90 days, yielding a mortality rate of 7.6% with a significant discrepancy between the non-MENA and MENA cohorts:45 (4.7%) vs. 75 (12.2%; *p* < 0.001) respectively.

**Figure 2:**
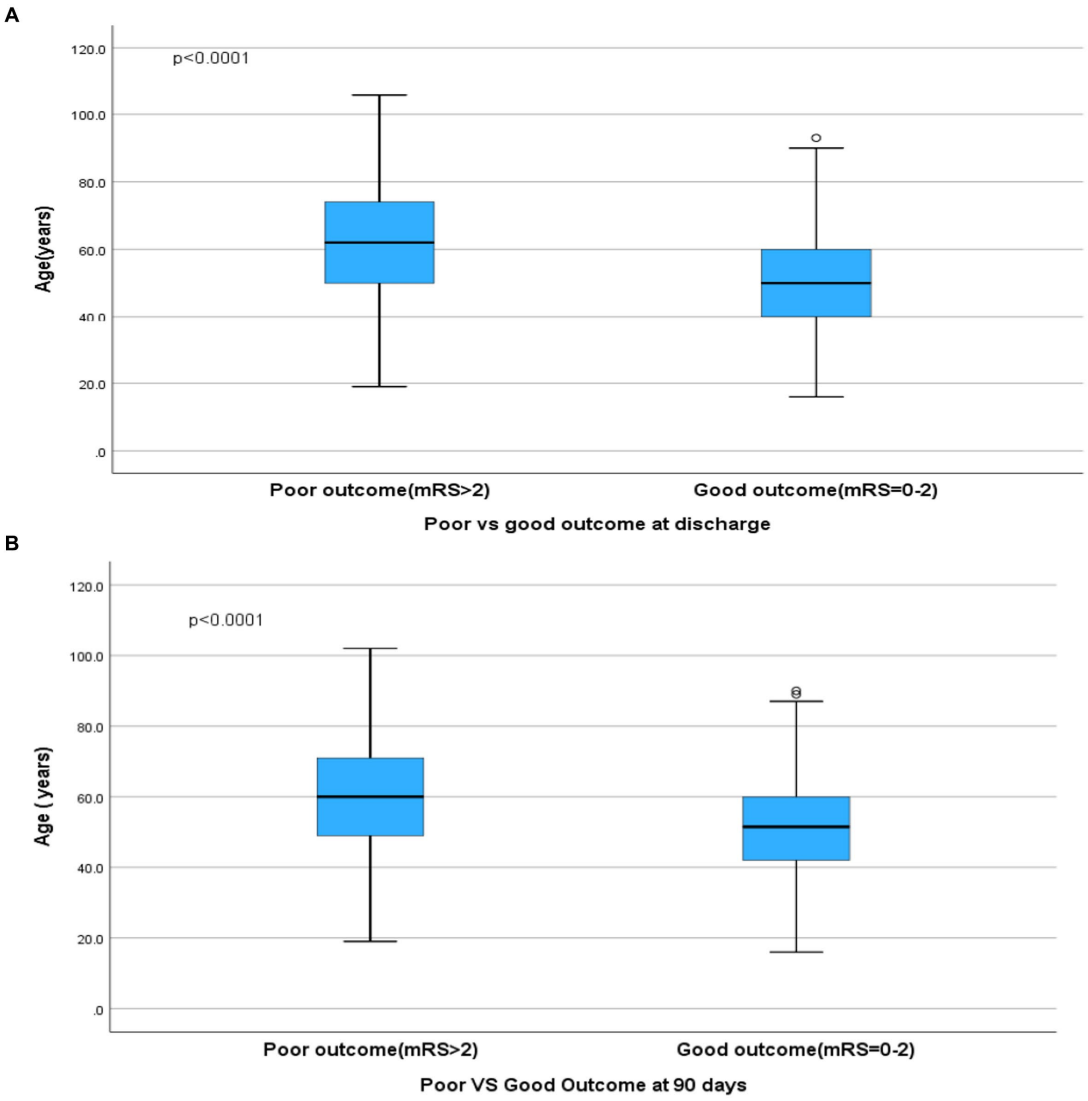
**(A,B)** Age and outcome at discharge and 90 days.

Dependency or death (mRS > 2) at discharge was seen in 621 (39.5%) patients and was significantly associated with increasing age (Figure 2), and proximal and multilocation involvement (*p* < 0.05).

**Figure 3:**
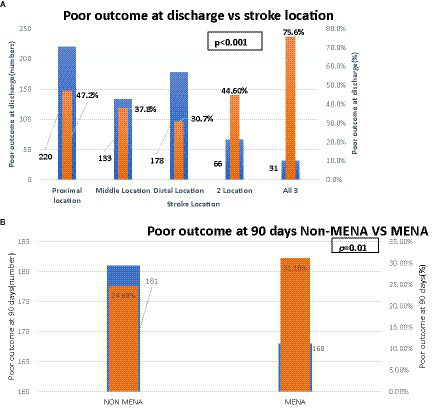
**(A)** Shows relationship between stroke location and poor outcomes at discharge. **(B)** Nationality and poor outcome at 90 days.

At 90 days, dependency or death was seen in 349/1,276 (27.4%) and was significantly associated with increasing age (Figure 2), male sex, and having a MENA nationality (*p* < 0.05; Figure 3).

## Discussion

In a large multiethnic group encompassing individuals from the Middle East and North Africa (MENA) and South Asia, noteworthy distinctions were noted between the younger non-MENA subgroup and the older MENA subgroup concerning the prevalence of vascular risk factors, stroke localization, mortality rates, and outcomes at 90 days. Our research aligns with prior studies indicating a high prevalence of vascular risk factors and favorable outcomes in PCS patients (32). Notably, we observed increased dependency or mortality rates in the older male MENA cohort. This contributes more insights into the non-white population data, thereby enhancing the existing literature that has traditionally been predominantly focused on white populations or characterized by limited ethnic diversity.

The incidence of PCS almost doubled from 2014 to 2019, coinciding with the establishment of organized stroke services, the launch of the stroke database, better case ascertainment with increased utilization of magnetic resonance imaging (MRI), and an increase in the population. The dip in 2020 coincides with the COVID-19 pandemic where stroke admissions were observed to have diminished (33).

### Demographics and clinical features

Compared to the historical NEMC-PCR study (6), our cohort showed similar age (average 61 years) and stroke location in the MENA group. In contrast, the South Asian non-MENA group was notably younger by a margin of 10 years, had a higher male-to-female ratio, and had a greater occurrence of proximal strokes. The higher representation of men in our cohort mirrors Qatar’s demographic skew toward men, primarily influenced by a significant presence of able-bodied South Asian blue-collar workers in the construction and gas sectors (34).

Both MENA and non-MENA groups in our study had high rates of vascular risk factors such as diabetes, hypertension, and dyslipidemia, aligning with findings from prior stroke cohorts in this region (17, 18) However, the older MENA group showed a higher incidence of CAD and AF. This emphasizes the necessity for effective screening and prevention programs at the primary health level.

Interestingly, the NEMC-PCR reported double the prevalence of CAD (35%) compared to our older MENA cohort. However, the incidence of AF was similar in both cohorts (34/407, 8.5%). It is noteworthy that the NEMC-PCR data was collected during a time when prolonged cardiac monitoring was not commonplace. As a result, there is a possibility that the prevalence of AF may have been underestimated, considering the significance of extended monitoring in identifying paroxysmal AF (35). Additionally, if taken at face value, the finding might suggest a lower prevalence of AF in the posterior circulation, as reported by Frid et al. (36).

The NIHSS scores were low in both the MENA and non-MENA cohorts, with a mean score of 3.8. This finding aligns with a large multicenter study conducted in Europe and North America (36) but is considerably lower than the scores reported in two Chinese cohorts (32, 37), 6.6 and 6.5, respectively. Low NIHSS scores in posterior circulation strokes are now widely recognized as a consequence of the scale’s limitations, primarily due to its focus on anterior circulation components (4).

### Stroke locations

Significant differences emerged between the MENA and non-MENA cohorts in stroke locations. The MENA group predominantly experienced distal strokes, followed by middle and proximal locations. Conversely, the non-MENA group had a higher frequency of proximal strokes, followed by distal and less frequently, middle locations (17, 18, 38).

This variation in stroke locations aligns with prior reports. Reports from predominantly white NEMC-PCR and a Chinese study (32) showed a similar prevalence of distal location strokes, whereas a Korean registry (38) highlighted middle PCS strokes as the most common. These differences in stroke locations might signal different underlying causes and potentially impact outcomes.

The predominance of distal location strokes in our population is likely due to small penetrating vessel disease (such as isolated thalamic lacunes) or artery-to-artery embolization from intracranial atherosclerosis, both prevalent stroke etiologies in our region, rather than cardioembolic strokes, as seen in reports from NEMC-PCR (17, 18, 20).

### Outcomes

Limited data exists on the overall prognosis of PCS, although it is believed to be similar to anterior circulation strokes when typical prognostic factors are accounted for (39).

The 90-day fatality rate in our report was influenced by the lower fatality rate in the non-MENA cohort that comprised 60% of the total cohort (4.7%), aligning with the low stroke mortality seen in Qatar. This was similarly reported in the NEMC-PCR (16, 40). Possible explanations would include younger age, the prevalence of small vessel etiology, and advanced stroke care in Qatar (41). Conversely, the MENA subgroup demonstrated nearly triple the mortality rate compared to both the non-MENA cohort and the NEMC-PCR, exceeding previously reported stroke mortality rates in Qatari patients (18). This dichotomy in mortality and dependence between the older, more vascularly burdened MENA cohort and the younger, less vascularly burdened non-MENA group underscores age as a consistent factor linked to poorer outcomes at both early and 90-day intervals.

Moreover, the MENA group, despite matching the NEMC-PCR in age, sex distribution, and prevalence of distal stroke location, showed higher fatality rates, suggesting the presence of other critical prognostic factors warranting further investigation.

Involvement of the proximal segment corresponds to worse short-term outcomes, echoing earlier findings associating cerebellar infarction and significant brainstem lesions with heightened mortality and morbidity (42). Similarly, multi-segmental involvement, likely linked to cardioembolic and vertebrobasilar thromboembolism, correlates with poorer outcomes (6, 32).

## Strength and limitations

The strength of this study lies in its origins as a prospective, extensive, and diverse dataset, uniquely established as a national database, thereby circumventing the constraints of conventional hospital-based registries. However, limitations of our research encompass the retrospective nature of data extraction and the presence of incomplete data at 90-day follow-up. Moreover, reliance on hospital-based registries is associated with potential errors in interpretation, documentation, and coding (43).

## Conclusion

We described the incidence, clinical features, and outcomes of PCS in a multiethnic cohort. Our study highlights the increase in incidence over time and the high prevalence of vascular risk factors and relatively poor outcomes in older male MENA patients which stresses the need for effective prevention and management of these patients. Further studies are needed to investigate potential genetic or environmental risk factors for PCS and their impact on outcomes in different populations.

## Data availability statement

The raw data supporting the conclusions of this article will be made available by the authors, without undue reservation.

## Ethics statement

The studies involving humans were approved by the Hamad Medical Corporation, Medical Research Center Institutional Review Board (research protocol 03-SI-17-075). The studies were conducted in accordance with the local legislation and institutional requirements. The ethics committee/institutional review board waived the requirement of written informed consent for participation from the participants or the participants' legal guardians/next of kin due to the retrospective nature of the study.

## Author contributions

YI: Conceptualization, Funding acquisition, Visualization, Writing – original draft, Writing – review & editing. PC: Formal analysis, Software, Validation, Writing – review & editing. RS: Formal analysis, Software, Validation, Writing – review & editing. IH: Data curation, Methodology, Project administration, Resources, Writing – review & editing. SAS: Methodology, Resources, Visualization, Writing – review & editing. MK: Resources, Writing – review & editing. NA: Investigation, Supervision, Writing – review & editing. SK: Investigation, Supervision, Writing – review & editing. SAJ: Investigation, Supervision, Writing – review & editing. AM: Investigation, Supervision, Writing – review & editing. KH: Investigation, Writing – review & editing. SH: Investigation, Writing – review & editing. JP: Formal analysis, Writing – review & editing. OE: Writing – review & editing. MAl: Writing – review & editing. LA: Investigation, Writing – review & editing. MAb: Writing – review & editing. SJ: Data curation, Writing – review & editing. DM: Data curation, Writing – review & editing. RU: Data curation, Writing – review & editing. ZB: Writing – review & editing. AAz: Writing – review & editing. AAy: Writing – review & editing. AE: Writing – review & editing. AO: Writing – review & editing. DD: Writing – review & editing.

## Funding

The author(s) declare that no financial support was received for the research, authorship, and/or publication of this article.
